# *Lactiplantibacillus plantarum* strains isolated from spontaneously fermented cocoa exhibit potential probiotic properties against *Gardnerella vaginalis* and *Neisseria gonorrhoeae*

**DOI:** 10.1186/s12866-021-02264-5

**Published:** 2021-06-29

**Authors:** Nathan das Neves Selis, Hellen Braga Martins de Oliveira, Hiago Ferreira Leão, Yan Bento dos Anjos, Beatriz Almeida Sampaio, Thiago Macêdo Lopes Correia, Carolline Florentino Almeida, Larissa Silva Carvalho Pena, Mariane Mares Reis, Thamara Louisy Santos Brito, Laís Ferraz Brito, Guilherme Barreto Campos, Jorge Timenetsky, Mariluze Peixoto Cruz, Rachel Passos Rezende, Carla Cristina Romano, Andréa Miura da Costa, Regiane Yatsuda, Ana Paula Trovatti Uetanabaro, Lucas Miranda Marques

**Affiliations:** 1 Programa de Pós-Graduação em Biologia e Biotecnologia de Microrganismos, Pavilhão Max de Menezes, Universidade Estadual de Santa Cruz, Campus Soane Nazaré de Andrade, Salobrinho, Rodovia Jorge Amado, Km 16, CEP 45662-900, Ilhéus, BA Brazil; 2 Instituto Multidisciplinar em Saúde, Universidade Federal da Bahia, Campus Anísio Teixeira, Rua Hormindo Barros, 58, CEP 45029-094, Vitória da Conquista, BA Brazil; 3 Instituto de Ciências Biomédicas, Universidade de São Paulo, Avenida Professor Lineu Prestes, 2415, CEP 05508-900, São Paulo, SP Brazil; 4 Departamento de Ciências Biológicas, Laboratório de Microbiologia da Agroindústria, Universidade Estadual de Santa Cruz, Campus Soane Nazaré de Andrade, Salobrinho, Rodovia Jorge Amado, Km 16, CEP 45662-900, Ilhéus, BA Brazil

**Keywords:** *L. plantarum*, *G. vaginalis*, *N. gonorrhoeae*, Cell-free culture supernatant, Organic acids, Probiotics, Metabolome

## Abstract

**Background:**

Probiotics are important tools in therapies against vaginal infections and can assist traditional antibiotic therapies in restoring healthy microbiota. Recent research has shown that microorganisms belonging to the genus *Lactobacillus* have probiotic potential. Thus, this study evaluated the potential *in vitro* probiotic properties of three strains of *Lactiplantibacillus plantarum*, isolated during the fermentation of high-quality cocoa, against *Gardnerella vaginalis* and *Neisseria gonorrhoeae*. Strains were evaluated for their physiological, safety, and antimicrobial characteristics.

**Results:**

The hydrophobicity of *L. plantarum* strains varied from 26.67 to 91.67%, and their autoaggregation varied from 18.10 to 30.64%. The co-aggregation of *L. plantarum* strains with *G. vaginalis* ranged from 14.73 to 16.31%, and from 29.14 to 45.76% with *N. gonorrhoeae*. All *L. plantarum* strains could moderately or strongly produce biofilms. *L. plantarum* strains did not show haemolytic activity and were generally sensitive to the tested antimicrobials. All lactobacillus strains were tolerant to heat and pH resistance tests. All three strains of *L. plantarum* showed antimicrobial activity against the tested pathogens. The coincubation of *L. plantarum* strains with pathogens showed that the culture pH remained below 4.5 after 24 h. All cell-free culture supernatants (CFCS) demonstrated activity against the two pathogens tested, and all *L. plantarum* strains produced hydrogen peroxide. CFCS characterisation in conjunction with gas chromatography revealed that organic acids, especially lactic acid, were responsible for the antimicrobial activity against the pathogens evaluated.

**Conclusion:**

The three strains of *L. plantarum* presented significant probiotic characteristics against the two pathogens of clinical importance. *In vitro* screening identified strong probiotic candidates for *in vivo* studies for the treatment of vaginal infections.

## Background

The misuse of antibiotics and the unavailability of newer drugs have been considered the main reasons for the current antimicrobial resistance crisis. In response to this, new approaches such as probiotics have shown promising results in trials, suggesting the role of alternative treatments as preventive or adjunct therapies in the future [1]. The Food and Agriculture Organization (FAO) defines probiotics as live microorganisms that confer a health benefit to the host when administered in adequate amounts [2]. In recent years, probiotic microorganisms have been investigated for their beneficial effects in many clinical studies on the treatment and prevention of various pathogens responsible for gastrointestinal disorders and vaginal infections in humans [3].

Vaginal infections are one of the main causes for gynaecological consultations [4]. In this study, two vaginal conditions were considered: bacterial vaginosis (BV) and gonorrhoea. BV is a dysbiosis common in adult women of reproductive age, characterised by the replacement of the resident microbiota composed of lactobacilli by various anaerobic bacteria, of which *G. vaginalis* is the most prevalent [5, 6]. With this dysbiosis, inflammation of the mucosa presents several symptoms, including vaginal discharge, itching, and burning associated with the lack of leukocytic exudate and redness. Other health complications related to BV include greater susceptibility to HIV infection, infections by specific microorganisms, pelvic inflammatory diseases, and premature births [7].

Gonorrhoea is a classic sexually transmitted infection caused by *N. gonorrhoeae*, a gram-negative intracellular diplococci bacterium [8]. Gonococcal infections can result in severe complications and sequelae, including pelvic inflammatory disease, infertility, ectopic pregnancy, first trimester abortion, and neonatal conjunctivitis, leading to blindness [9]. Within this panorama, it is clear that as gonorrhoea causes great morbidity and has significant socioeconomic consequences, the emergence of therapies to reduce gonococcal infections without the use of antibiotics would be beneficial for public health worldwide [10].

In this context, it is understood why research on probiotics has been successful in therapies for women's health. Probiotic properties have been observed in many genera of bacteria and fungi, but the most commonly used probiotics belong to the genus *Lactobacillus*, particularly to the species *L. plantarum*. Historically, lactobacilli have been generally recognised as safe (GRAS) for consumption and therapeutic applications [11].

*L. plantarum* has been widely used as a model species for ecological, metabolic, and genetic studies. Furthermore, *L. plantarum* is of commercial importance as a starter culture for multiple food fermentations, and is used as a probiotic culture [12].

Many lactobacilli strains are able to colonise and produce antimicrobials that prevent the growth of pathogenic microorganisms [13]. From this perspective, the search for new probiotics is motivated by the knowledge that each lactobacillus strain possesses different properties and could have unique effects on human health. A few studies have reported on lactobacilli strains isolated from non-human sources that have shown promising probiotic effects [14–16]. In the present study, we evaluated the *in vitro* probiotic potential of three strains of *L. plantarum,* isolated during the fermentation of high-quality cocoa, against two vaginal pathogens, *G. vaginalis* and *N. gonorrhoeae*.

## Results

### Cell surface properties of *Lactobacillus*

Results of hydrophobicity (H%), autoaggregation (AA%), co-aggregation (CA%), and biofilm formation of the *Lactobacillus* strains are shown in Table 1. The hydrophobicity of the *Lactobacillus* strains ranged from 26.67 to 91.67%. Among the three *Lactobacillus* strains, only the Lp289 strain showed a significant increase in hydrophobicity compared to the Lp291 and Lp03 strains (*P* < 0.05). Regarding the first classification that considers the microbial adhesion to hydrocarbons, the Lp03 strain showed moderate hydrophobicity, Lp289 strain showed high hydrophobicity, and Lp291 strain showed low hydrophobicity. According to the second classification that considers microbial adhesion to solvents, Lp03 and Lp291 strains were considered hydrophilic, while *L. plantarum* Lp289 was considered hydrophobic.

**Table 1:** Characterization of physiological properties and haemolytic activity of *Lactobacillus* strains isolated from cocoa fermentation

Strain	Hydrophobicity (%)	Autoaggregation (%)	Coaggregation (%)		Biofilm formation	Haemolytic activity
			*G. vaginalis*	*N. gonorrhoeae*		
Lp03	36.93 ± 2.91^a^	22.22 ± 1.67^a^	16.31 ± 0.42^a1^	45.76 ± 1.49^a2^	Strongly adherent	γ-haemolytic
Lp289	91.67 ± 1.49^b^	18.10 ± 0.83^a^	18.12 ± 3.62^a1^	29.14 ± 2.99^b2^	Moderately adherent	γ-haemolytic
Lp291	26.67 ± 3.34^a^	30.64 ± 3.48^b^	14.73 ± 1.11^a1^	32.84 ± 4.08^b2^	Moderately adherent	γ-haemolytic

Presented values are means of triplicate determinations; ± indicates standard deviations from the mean. Mean values (±standard deviation) within the same column followed by different superscript letters are statistically significant (*P* < 0.05). In coaggregation, mean values (±standard deviation) within the same line followed by different superscript numbers were statistically significant (*P* < 0.05)

An autoaggregation assay was performed to evaluate the ability of *Lactobacillus* to aggregate with strains of the same species. Autoaggregation rates obtained for *Lactobacillus* ranged from 18.10 to 30.64% after 5 h of incubation. The highest level of autoaggregation was observed for *L. plantarum* Lp291 (30.64%), which differed significantly from the Lp289 and Lp03 strains (*P* < 0.05).

Similarly, a co-aggregation assay was performed to evaluate whether *Lactobacillus* strains interact directly with genital pathogens. All *Lactobacillus* strains co-aggregated with the applied vaginal pathogens, with distinct levels of interaction, and exhibited a higher percentage of co-aggregation with *N. gonorrhoeae* compared to *G. vaginalis* (*P* < 0.05)*.* However, there was no significant difference in the co-aggregation of *Lactobacillus* strains with *G. vaginalis* or with *N. gonorrhoeae* (*P* > 0.05). In addition, our data demonstrated that the lactobacilli strains could adhere and form biofilms under the conditions tested. *L. plantarum* Lp03 was strongly adherent, and Lp298 and 291 strains were moderately adherent.

### Evaluation of haemolytic activity and antibiotic susceptibility of *Lactobacillus* strains

In the present study, none of the *Lactobacillus* strains showed haemolytic activity. Results described in Table 1 demonstrate that the Lp03, Lp289, and Lp291 strains were considered γ-haemolytic. Together with these data, results in Table 2 demonstrate the susceptibility profile of *Lactobacillus* strains to antibiotics based on the disk diffusion method. Nine antibiotics belonging to different classes were used in this study, including cell wall, protein, and nucleic acid synthesis inhibitors, and urinary tract antiseptics. Results of this trial revealed that the three strains of *L. plantarum* demonstrated resistance to vancomycin only. The data also showed that *L. plantarum* Lp03 and Lp289 were susceptible to increased exposure to ciprofloxacin. All strains were classified as sensitive to other antibiotics. The Lp03 and Lp289 strains demonstrated identical antimicrobial susceptibility profiles.

**Table 2:** Antimicrobial susceptibility profile of *Lactobacillus* strains isolated from cocoa fermentation

Antimicrobial			Susceptibility			
Type	Name	Disc contents	Lp03	Lp289	Lp291	*S. aureus* ATCC 25923
Inhibitors of cell wall synthesis	Ampicillin	10 μg	S	S	S	SIE
	Ceftriaxone	30 μg	S	S	S	S
	Penicillin G	10 μg	S	S	S	R
	Vancomycin	30 μg	R	R	R	S
Inhibitors of protein synthesis	Clindamycin	2 μg	S	S	S	S
	Chloramphenicol	30 μg	S	S	S	S
	Erythromycin	15 μg	S	S	S	SIE
Inhibitors of nucleic acid synthesis	Ciprofloxacin	5 μg	SIE	SIE	S	S
Other urinary tract antiseptics	Nitrofurantoin	300 μg	S	S	S	S

Susceptibility is expressed as sensitive (S), susceptible, increased exposure (SIE), or resistant (R) [17]. *Staphylococcus aureus* ATCC 25923 was used as the positive control

### Evaluation of the resilience of lactobacilli strains to thermal and pH stress

**Fig. 1:**
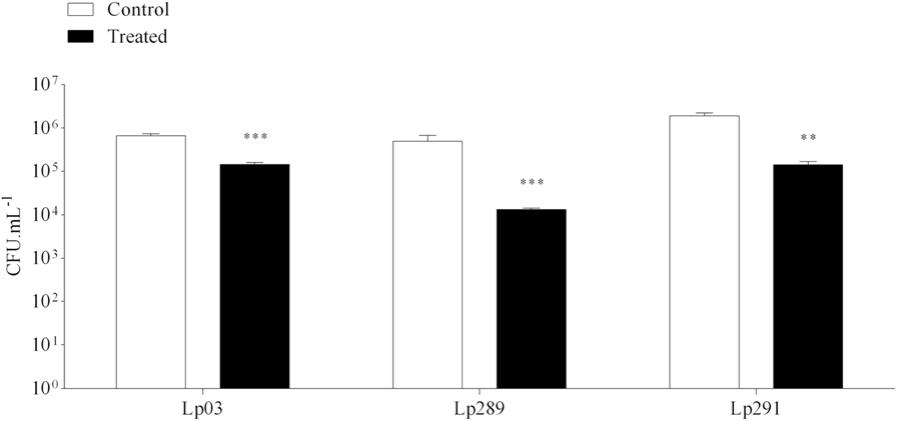
Heat resistance standard of *Lactobacillus* strains isolated from cocoa fermentation. (Lp03) *L. plantarum* Lp03; (Lp289) *L. plantarum* Lp289; (Lp291) *L. plantarum* Lp291. (**) Statistically significant differences compared to control (*P* < 0.01); (***) Statistically significant differences compared to control (*P* < 0.001). Presented values represent the mean and standard deviation from triplicate determinations

**Fig. 2:**
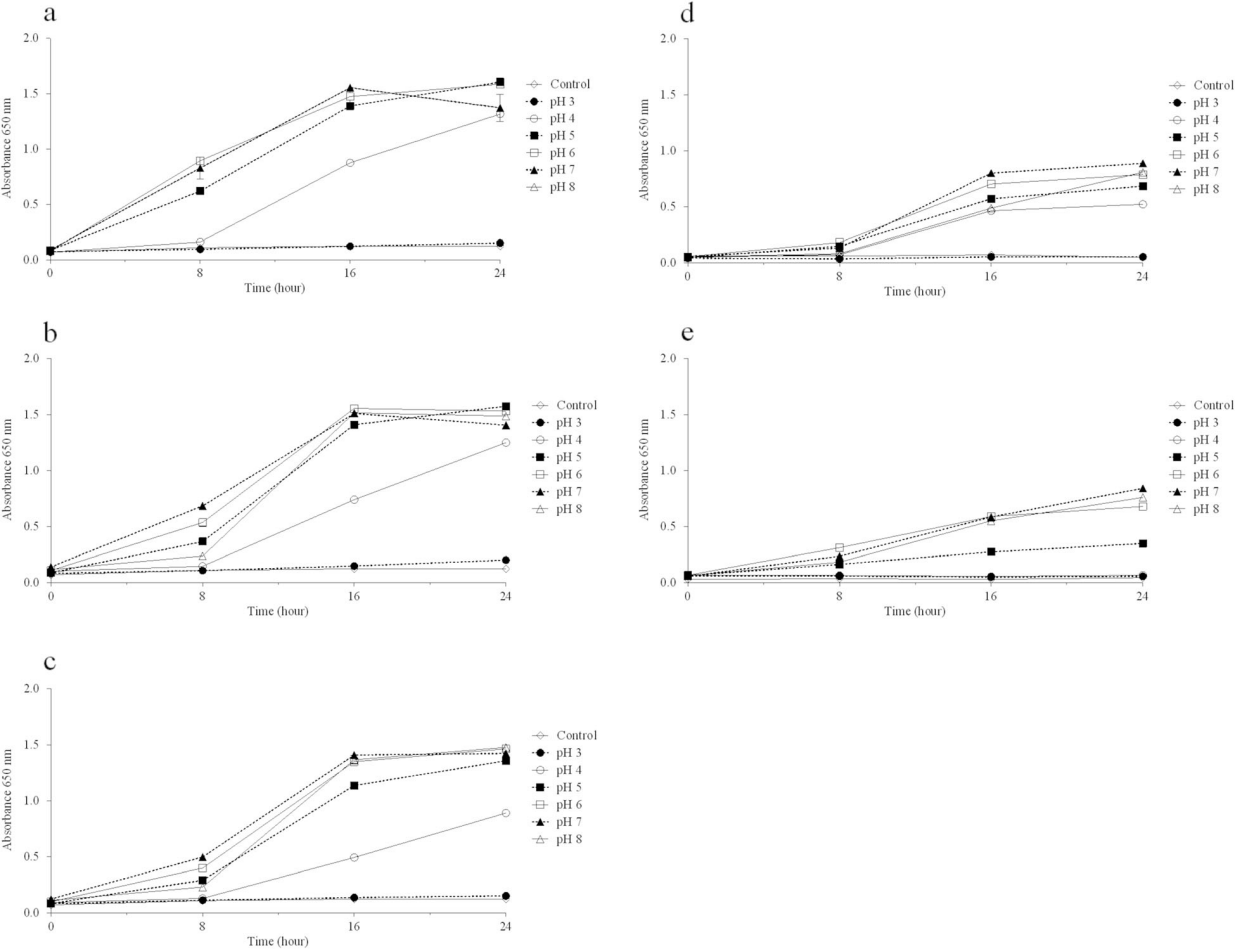
Growth of *Lactobacillus* strains and pathogens in different pH ranges. **a** *L. plantarum* Lp03; **b** *L. plantarum* Lp289; **c** *L. plantarum* Lp291; **d** *G. vaginalis*; **e** *N. gonorrhoeae*. Presented values represent the mean and standard deviation from triplicate determinations

Concerning thermal resistance of *Lactobacillus* strains, there was a significant reduction (*P* < 0.05) in the viable cell counts of the three strains after thermal shock, as can be seen in Fig. 1. Regardless, all lactobacilli strains remained viable after treatment. *L. plantarum* tested were also evaluated for their ability to grow in different pH ranges, ranging from acidic to basic environments. Results of this assay are shown in Fig. 2. Our data showed that all *Lactobacillus* strains grew at all pH values, except at pH 3. The same pH tolerance assay was applied to the pathogens to characterise their growth profiles. Similar to *Lactobacillus* strains*, the G. vaginalis* strain grew at all pH values, except at pH 3. For the *N. gonorrhoeae* strain, the pathogen was able to grow only in media with a pH above 5.

### Inhibition of pathogen growth by lactobacilli strains

**Fig. 3:**
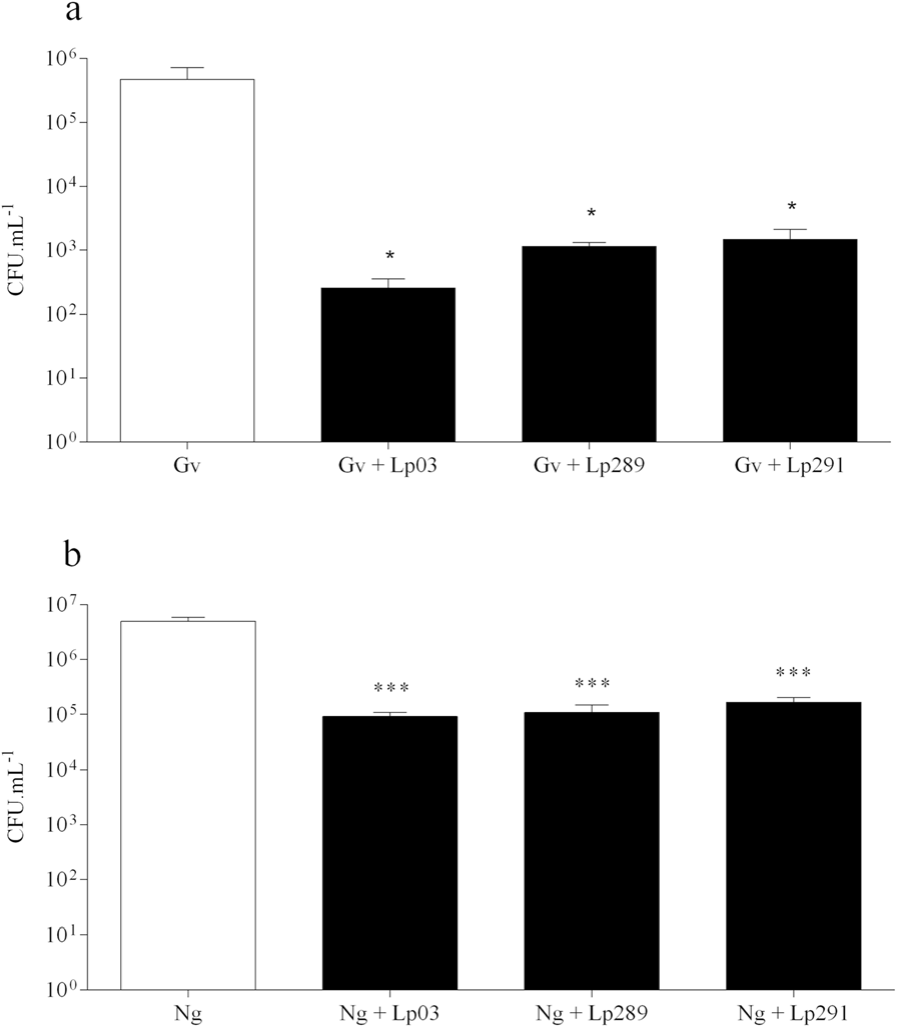
Growth inhibition of pathogens by *Lactobacillus* strains after 24 h of culture. The growth of pathogenic microorganism is expressed as log_10_ CFU mL^−1^. Control: (Gv) *G. vaginalis* or (Ng) *N. gonorrhoeae*. The different *Lactobacillus* strain isolates are represented by their respective numbers. **a** represents coculture of *G. vaginalis* with *Lactobacillus* sp.; **b** represents coculture of *N. gonorrhoeae* with *Lactobacillus* sp. (*) Statistically significant differences compared to control (*P* < 0.05); (***) Statistically significant differences compared to control (*P* < 0.001). Presented values represent the mean and standard deviation from triplicate determinations

Co-incubation experiments were performed to quantitate growth inhibition of *G. vaginalis* and *N. gonorrhoeae* to evaluate the possible inhibition caused by a direct interaction between *Lactobacillus* strains and pathogen cells. The inhibitory effects of the lactobacilli strains against the tested pathogens are shown in Fig. 3. Our data showed that all *Lactobacillus* strains inhibited both *G. vaginalis* and *N. gonorrhoeae* when co-incubated. After 24 h of co-incubation, results showed that all *Lactobacillus* strains were able to significantly reduce the viability of *G. vaginalis* (*P* < 0.05) and *N. gonorrhoeae* (*P* < 0.001) with different levels of effectiveness. There was a significant reduction in the CFU mL^-1^ count of pathogens compared to monoculture (10^6^), with counts of approximately 10^2^ CFU mL^-1^ and 10^5^ CFU mL^-1^ for *N. gonorrhoeae* and *G. vaginalis*, respectively.

### Evaluation of pH reduction by lactobacilli in cultures with or without pathogen

The pH of the bacterial growth medium was measured in isolated cultures of *Lactobacillus* strains and in co-culture of lactobacilli with pathogens (Table 3). The lowest pH recorded was from the isolated culture of the Lp03 strain (3.76), and the highest pH was observed in the co-culture of the Lp291 strain with *N. gonorrhoeae* (4.47). There were no significant differences (*P* > 0.05) between the pH of the growth media of the *Lactobacillus* strains growing alone or in combination with the pathogens. This demonstrated that the pH did not change in the presence of the pathogens tested. We also observed that the three *Lactobacillus* strains were able to reduce the pH of the culture medium from an initial pH of 6.5, to values below 4.5, in all culture conditions.

**Table 3:** Antimicrobial evaluation of *Lactobacillus* strains and CFCS against *G. vaginalis* and *N. gonorrhoeae*

Strain/Medium	pH			Deferred inhibition (mm)		Microdiffusion (mm)	
	Isolated growth	Coincubation (Gv)	Coincubation (Ng)	*G. vaginalis*	*N. gonorrhoeae*	*G. vaginalis*	*N. gonorrhoeae*
Lp03	3.76 ± 0.08^a1^	3.85 ± 0.03^a1^	3.85 ± 0.02^a1^	19.67 ± 2.52	32.67 ± 2.52	Contact	11.33 ± 3.06
Lp289	3.85 ± 0.01^a1^	3.86 ± 0.02^a1^	3.82 ± 0.02^a1^	15.33 ± 3.06	23.33 ± 5.13	18.33 ± 2.08	18.67 ± 1.53
Lp291	3.92 ± 0.10^a1^	4.23 ± 0.01^a1^	4.47 ± 0.01^a1^	20.67 ± 1.16	26.33 ± 0.58	12.33 ± 1.53	Contact
MRS	6.50 ± 0.10^b1^	6.50 ± 0.10^b1^	6.50 ± 0.10^b1^	0.00 ± 0.00	0.00 ± 0.00	0.00 ± 0.00	0.00 ± 0.00

pH assay values are means of triplicate determinations; ± indicates standard deviations from the mean. Mean values (± standard deviation) within the same column followed by different superscript letters are statistically significant (*P* < 0.05). Mean values (±standard deviation) within the same line followed by different superscript numbers were statistically significant (*P* < 0.05). (Gv) *G. vaginalis*; (Ng) *N. gonorrhoeae*. The measurement of the inhibition halos of the microdiffusion assay included the diameter of the PVC cylinder (8 mm). Contact means that there was no pathogen growth inside the PVC cylinder

### Identification and characterization of the antimicrobial activity of CFCS against pathogens

Tables 3 and 4 show the effects of *Lactobacillus* strains and their CFCS on *G. vaginalis* and *N. gonorrhoeae*, and the effect of different physical and chemical treatments of CFCS on antimicrobial activity, respectively. The inhibitory activity of the *Lactobacillus* strains against vaginal pathogens was assessed through two different *in vitro* experiments. Our data demonstrated that bioactive compounds produced by *Lactobacillus* strains inhibited the growth of *N. gonorrhoeae* and *G. vaginalis*, inducing the formation of an inhibition halo around the colony. All strains had greater inhibition halos for *N. gonorrhoeae* than *G. vaginalis*.

**Table 4:** Antimicrobial activity of treated and untreated CFCS of *Lactobacillus* strains against *G. vaginalis* and *N. gonorrhoeae*

Strain	MRS	Untreated CFCS	Neutralized CFCS	Boiled CFCS	CFCS + Trypsin	CFCS + Proteinase K
**Growth of** ***G. vaginalis***						
Lp03	+	Inhibited	+	Inhibited	Inhibited	Inhibited
Lp289	+	Inhibited	+	Inhibited	Inhibited	Inhibited
Lp291	+	Inhibited	+	Inhibited	Inhibited	Inhibited
**Growth of** ***N. gonorrhoeae***						
Lp03	+	Inhibited	+	Inhibited	Inhibited	Inhibited
Lp289	+	Inhibited	+	Inhibited	Inhibited	Inhibited
Lp291	+	Inhibited	+	Inhibited	Inhibited	Inhibited

(+) indicates pathogen growth

Results of the microdiffusion assay showed that the bioactive compounds inhibited the growth of pathogens, showing inhibition by contact or forming halos of moderate inhibition. Contact inhibition refers only to the area of the agar within the PVC cylinder, which maintained direct contact with CFCS and did not show growth of pathogens. Only the Lp298 strain was able to inhibit both pathogens with similarly sized halos.

The Lp03 strain was able to inhibit *N. gonorrhoeae* more effectively than *G. vaginalis,* and the Lp291 strain was able to inhibit *G. vaginalis* more effectively than *N. gonorrhoeae*.

After this verification, a characterisation test of the antimicrobial substances present in CFCS was carried out. Results presented in Table 4 reveal the inhibitory activity of the treated and untreated CFCS of *Lactobacillus* strains against *G. vaginalis* and *N. gonorrhoeae*. Treatments applied to the CFCS of each *Lactobacillus* strain were established to identify which substances were responsible for the inhibition of pathogens in the previous tests. Treatments of CFCS included neutralisation of organic acids, boiling, and inactivation of possible bacteriocins through the enzymatic action of trypsin or proteinase K. Our data demonstrated that both pathogens had the same inhibition response with respect to CFCS treated or not in this trial. The CFCS boiled or treated with trypsin or proteinase K did not affect their inhibitory activities against the two pathogens tested.

**Fig. 4:**
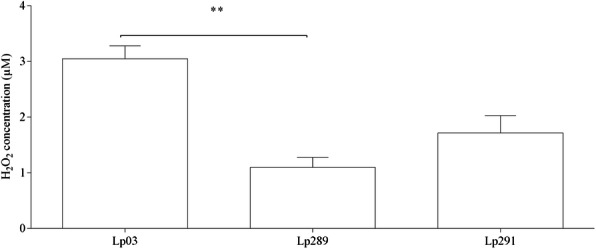
Hydrogen peroxide levels present in CFCS of *Lactobacillus* strains isolated from cocoa fermentation. The standard curve (R^2^ = 0.9927) was performed together with the experimental samples in a controlled environment protected from light. Presented values represent the mean and standard deviation from triplicate determinations. (**) Statistically significant differences (*P* < 0.01)

In addition to these compounds, we identified that the *Lactobacillus* strains were hydrogen peroxide producers; all strains were able to produce hydrogen peroxide at concentrations varying between and 1-5 μM, as shown in Fig. 4. The Lp03 strain showed higher H_2_0_2_ production than the Lp289 strain (*P* < 0.01), and there was no significant difference in hydrogen peroxide production in the Lp291 strain compared to the other two strains (*P* > 0.05).

### CFCS metabolome profile

The metabolome of *Lactobacillus* strains proved to be diverse, with variability of substances such as organic acids, alcohols, sugars, and other organic compounds. The present study specifically evaluated the metabolome of *Lactobacillus* strains for organic acid production, as shown in Table 5. In this regard, we observed that lactic acid was the most abundant organic acid produced by all *Lactobacillus* strains, constituting more than 50% of the CFCS sample. The Lp03 strain had the highest percentage of lactic acid (68.16%) among all samples, followed by phosphoric acid (7.18%) and 1,2,3-propanetricarboxylic acid (4.27%). Following the same pattern, the main organic acids produced by the Lp289 strain were lactic acid (56.35%), phosphoric acid (8.32 %), 1,2,3-propanetricarboxylic acid (4.33%) and butyric acid (1.53%). The Lp291 analysis of the CFCS strain revealed the prevalence of lactic acid (67.34%), phosphoric acid (7.92%), acetic acid (6.52%), and 1,2,3-propanetricarboxylic acid (3.34%).

**Table 5:** Metabolomic analysis of CFCS of *Lactobacillus* strains Lp03, Lp289 and Lp291

Retention time (min)	Substance	MRS area (%)	CFCS area (%)	CFCS area (%)	CFCS area (%)
			Lp03	Lp289	Lp291
4.164	Carbodiimide	–	1.69	1.70	–
4.700	N.N-Dimethylglycine	0.04	–	0.15	0.09
7.295	Lactic acid	0.85	68.16	56.35	67.34
7.524	Acetic acid	–	–	0.07	6.52
7.768	Valine	0.05	–	0.04	1.02
7.936	2-Propenoic acid	–	–	0.05	0.11
8.405	Alanine	1.14	1.74	2.38	–
9.020	Glycine	0.33	0.76	1.11	–
9.873	β- Lactate	–	–	0.08	–
10.168	Leucine	0.17	0.16	0.07	1.29
10.445	3-Hydroxybutyric acid	–	–	0.04	0.26
10.638	α-Hydroxyvaleric acid	–	0.12	0.16	–
10.909	Isoleucine	–	–	–	0.79
12.465	Valine	1.02	1.69	2.41	0.15
13.203	4-methyl-2-hydroxypentanoic acid	–	0.41	0.52	0.58
13.395	3-methyl-2-hydroxypentanoic acid	–	0.12	0.08	0.06
14.510	Leucine	2.48	2.65	3.79	–
14.733	Glycerol	–	0.95	–	–
14.769	Phosphoric acid	7.29	7.18	8.32	7.92
15.236	Isoleucine	1.18	1.59	2.44	0.44
15.308	γ- Amino butyric acid	–	–	0.03	–
15.839	Butanoic acid	0.20	0.43	0.57	0.41
16.220	2-methyl-2.3-dihydroxypropanoic acid	0.10	0.16	0.20	0.28
16.705	Pyrimidine	–	–	0.05	–
17.644	Serine	0.75	0.25	0.64	0.61
18.148	Butanoic acid	0.02	0.43	0.09	0.08
18.272	3-Methyl-1.4-dihydroxypiperazine-2.5-dione	–	–	0.02	–
18.496	Butyric acid	–	0.49	1.53	–
18.531	Threonine	0.62	–	–	–
18.555	Lactic acid dimer	–	0.33	0.26	0.77
19.332	2.4-dihydroxybutanoic acid	–	–	0.08	0.08
21.091	Trisiloxane	–	0.13	0.12	0.09
21.769	Malic acid	–	–	0.07	0.09
21.865	2-Pyrrolidone-5-carboxylic acid	–	0.33	0.41	0.41
22.262	Glutamic acid	–	–	0.05	–
22.458	Methionine	0.22	–	–	–
22.588	Proline	1.40	0.89	2.90	0.35
22.708	Aspartic acid	0.56	–	0.55	–
23.099	Phenylalanine	–	0.79	–	0.44
24.376	Benzenepropanoic acid	–	0.36	0.53	0.12
25.583	Glutamine	1.76	–	2.79	–
26.434	Tartaric acid	0.34	0.35	0.51	–
26.995	Hydroxy 4-oxo-2.4-di(hydroxyamino)butanoate	–	–	–	0.20
27.048	Asparagine	0.04	–	–	–
27.895	Lysine	–	–	0.63	–
28.638	Arabinitol	–	–	0.23	–
28.805	Ribitol	–	0.17	–	–
29.803	2.3-dihydroxypropylphosphoric acid	0.13	–	0.24	–
29.920	D-Ribo-Hexonic acid	–	–	0.09	–
30.946	2-Keto-D-gluconic acid	0.70	–	0.10	–
31.133	1.2.3-Propanetricarboxylic acid	–	4.27	4.33	3.34
31.259	Citric acid	7.49	–	–	–
32.345	Pentanedioic acid	–	0.23	0.42	0.10
32.451	Benzenepropanoic acid	0.10	–	–	–
32.879	4-Hydroxyphenyllactic acid		0.12	–	–
33.891	Tyrosine	0.13	–	0.05	–
33.955	Glucitol	0.04	–	–	0.18
35.660	Inositol	31.07	–	–	–
39.845	Tryptophan	0.06	–	0.07	–
44.979	Uridine	–	–	–	0.09
–	Identified compounds (except sugars)	60.28	96.94	97.32	94.34
–	Sugars	38.12	1.14	2.31	5.28
–	Unidentified compounds	1.60	1.92	0.37	0.38
–	Total	100.00	100.00	100.00	100.00

In addition, trace amounts of other organic acids, including 3-hydroxybutyric, α-hydroxyvaleric, 4-methyl-2-hydroxypentanoic, 3-methyl-2-hydroxypentanoic, butyric, 2.4-dihydroxybutanoic, malic, 2-pyrrolidone-5-carboxylic, and benzenepropanoic acids. Although acid production is strain-specific, we observed that γ-amino butyric, glutamic, aspartic, 2.3-dihydroxypropylphosphoric, D-ribo-hexonic, and 2-keto-D-gluconic acids were detected only in samples of the Lp289 strain. In the present study, we observed that the prominent presence of phosphoric acid in the samples was due to the MRS medium.

## Discussion

Cell surface hydrophobicity is an important characteristic of potential probiotics as it indicates whether *Lactobacillus* strains can bind to the mucosa. This mechanism is one of the major factors by which probiotic bacteria are believed to exert beneficial effects in the host [18]. It is known that a large variety of surface glycoproteins are inserted into the hydrophobic cell wall of some microorganisms and they are responsible for increasing the likelihood of adhesion to cell receptors or proteins anchored in the cell wall [19–21]. Based on literature, we observed that strain 289 had a potential common to probiotics because of its high hydrophobicity. In addition, as the *Lactobacillus* strains were isolated from cocoa fermentation and that the cocoa pulp is made up of 82 to 87% of water, this could explain the low hydrophobicity of the Lp03 and Lp291 strains [22].

In addition to hydrophobicity, autoaggregation is another probiotic criterion allowing the colonisation and adherence of bacteria to epithelial cells, leading to the prevention of colonisation by pathogens, one of the main defence mechanisms against infection of the urogenital tract [23]. Our data revealed that the three strains of *L. plantarum* had a low percentage of autoaggregation. *Lactobacillus* strains usually show an autoaggregation capacity ranging from low to moderate [24], indicating that results are within the expected range, as there is great variation in autoaggregation among strains of both human vaginal microbiota and nonhuman origin [17]. Other studies have demonstrated that the values of autoaggregation are quite variable within the same species or genus *Lactobacillus*, which does not preclude the use of lactobacilli with low autoaggregation as probiotics [25–28].

Another important tool that demonstrates *Lactobacillus* strains use to eliminate bacteria is the ability to aggregate pathogens. Co-aggregation is one of the mechanisms exerted by probiotics to create a competitive microenvironment around the pathogen [29]. In our study, all strains co-aggregated more effectively with *N. gonorrhoeae* than with *G. vaginalis*. Similarly, Vielfort et al. [30] reported that their lactobacilli strains exhibited the ability to interact and aggregate with *N. gonorrhoeae*, configuring this process as an important mechanism to neutralise gonococci viability. This close interaction permits the *Lactobacillus* strains to create an unfavourable microenvironment for pathogens, where antimicrobial substances produced by lactobacilli in a localised manner harm epithelial colonisation by pathogens [31].

Biofilm formation by lactobacilli can be considered a determinant element for a probiotic microorganism because it is important to promote colonisation and persistence of *Lactobacillus* strains on vaginal epithelium, and to exert their protective role by interfering with the growth and adhesion of pathogens [32]. Kaur et al. [33] reported that the maturation of biofilms is strongly dependent on the autoaggregation properties of the probiotic microorganism, as it helps the bacteria to form micro-colonies. However, this relationship was not observed in the present study. All *Lactobacillus* strains were able to adhere to the abiotic polystyrene device, and the Lp03 strain stood out as a strong biofilm producer. The methodology used in our study showed that all strains tested were able to moderately or strongly form biofilms on polystyrene surfaces [34].

The absence of haemolytic activities presented by our lactobacilli strains is a recommended safety characteristic in probiotic selection [31, 32]. Regarding the phenotypical vancomycin resistance, it is noteworthy that most of *Lactobacillus* sp. are intrinsically resistant to this antibiotic [26, 35]. The gene responsible for this resistance is chromosomal, and therefore cannot be transferred by mobile genetic elements to other bacteria. Probiotics with this type of antimicrobial resistance are already used to restore the microbiota after treatment with antibiotics without posing a risk to human health [36, 37].

In addition to the safety characteristics of the strains, it is interesting that lactobacilli have been approved for resilience tests. It is known that during different industrial and biotechnological processes, probiotic bacteria must respond rapidly to stress to survive, and heat is among the most destructive stress conditions. Exposure to high temperatures destabilises macromolecules such as RNA and ribosomes, leading to denaturation of proteins and alterations in membrane fluidity, which have also been reported [38]. As our strains were isolated from cocoa fermentation, we propose that the tested strains are similarly adapted to the relatively high temperature stress found during spontaneous cocoa fermentation which can reach 50°C [39].

Under the influence of oestrogen, glycogen is deposited in the human vagina, and the *Lactobacillus* strains use this glycogen to produce lactic acid. Thus, acidification of the vagina (pH ≤ 4.5) results in growth inhibition of other bacteria [40, 41]. Since vaginal pathogens colonise the vagina and raise the pH (4.5 to 6.0), these *Lactobacillus* strains can be used under these situations to acidify the mucosa and displace the pathogens, assisting other types of interventions such as antibiotic therapy [42, 43].

As observed in our work, many authors have reported that some *Lactobacillus* strains have the ability to inhibit *G. vaginalis* and *N. gonorrhoeae* growth [44–46]. The co-culture assay is able to assess the influence of one microorganism on the growth of another when both are incubated together, simulating what actually happens in the vaginal environment [47]. The growth-inhibiting activity of lactobacilli has generally been attributed to its lowering of pH, and production of lactic acid, hydrogen peroxide, and antibacterial compounds [48]. It is interesting to note that in each co-culture of *Lactobacillus* strains with pathogenic strains, the pH remained below 4.5, reinforcing both the presence of organic acids produced by *L. plantarum* strains and an acidic environment common to the healthy vagina [34].

Literature often correlates the probiotic activity of *Lactobacillus* strains against vaginal pathogens with the production of metabolites such as hydrogen peroxide [49]. Bacterial cell membranes are known to be semipermeable to H_2_O_2_, a reactive oxygen species, and act intracellularly, forming free radicals that cause widespread damage to DNA, membranes, enzymes, and proteins [50]. However, although vaginal colonisation by hydrogen peroxide-producing *Lactobacillus* strains is associated with lower rates of bacterial vaginosis, some authors suggest that the presence of these lactobacilli alone is not able to suppress BV-associated infection [51], and that lactic acid is the true effector molecule against uropathogens [52, 53].

The identification and characterisation of the antimicrobial substances produced by *Lactobacillus* strains demonstrated that organic acid was the key molecule in inhibiting both pathogens. In accordance with our data, Shokryazdan et al. [54] observed that the antimicrobial activity present in the CFCS of *Lactobacillus* strains was due to organic acids and antibacterial substances which can inhibit microbial growth by lowering the pH [55]. The physiological importance of lactic acid has been well documented in a review by Tachedjian et al. [56], which reported that lactic acid at physiological concentrations (110 mM), even at pH 4.5, mediates a potent 10^6^-fold decrease in the viability of 17 different BV-associated microorganisms, but does not affect the viability of four vaginal lactobacilli *in vitro*. Lactic acid also acts not only on pathogens, but also interferes with vaginal immunomodulation, by directly inhibiting pro-inflammatory responses by IL-6, IL-8, and IL-1RA and inducing the Th17 lymphocyte pathway via IL-23 [57], promoting vaginal tissue homeostasis.

## Conclusion

This study showed the potential probiotic characteristics of *L. plantarum* 03, *L. plantarum* 289, and *L. plantarum* 291 against *G. vaginalis* and *N. gonorrhoeae*. *L. plantarum* strains isolated from cocoa fermentation are safe and have probiotic properties, including biofilm formation, tolerance to heat and pH, direct competition with pathogens, and production of lactic acid and hydrogen peroxide. Our results indicate that these three lactobacilli strains have desirable properties for the development of future therapeutic agents; however, *in vivo* studies are necessary to confirm their properties against the tested vaginal pathogens.

## Methods

### Microorganisms and growth conditions

Three strains of *Lactiplantibacillus plantarum* (Lp03, Lp289, and Lp291) were isolated from spontaneous cocoa (*Theobroma cacao*) from the region of Ilhéus and Itabuna, BA, Brazil, and donated by the Laboratory of Applied Microbiology from the State University of Santa Cruz, Ilhéus, BA, Brazil [22]. Lactobacilli strains were grown in de Man, Rogosa, and Sharpe (MRS) agar or broth (Acumedia, Lansing, USA) for 18 to 24 h, at 37°C under microaerophilic conditions (5% CO_2_ atmosphere).

*Gardnerella vaginalis* ATCC 49154 was grown on 5% blood agar (HiMedia, Mumbai, India) or brain and heart infusion (BHI) (HiMedia) for 18 to 24 h at 37°C under microaerophilic conditions (5% CO_2_ atmosphere).

*Neisseria gonorrhoeae* (clinical isolate) was grown on chocolate agar (HiMedia) or BHI for 18 to 24 h, at 37°C under microaerophilic conditions (5% CO_2_ atmosphere).

### Preparation of cell-free culture supernatant

The assay for obtaining CFCS was adapted from Pessoa et al. [47]. After overnight cultures of *Lactobacillus* strains were centrifuged (15 min, 3000 *×g*), supernatants were discarded, and cell pellets (lactobacilli) were washed twice with sterile saline (0.9% NaCl) and resuspended to a final cell density (10^8^ CFU mL^-1^). Suspensions (1,5 mL) of each *Lactobacillus* strain were then inoculated (10%, v/v) in sterile MRS broth (15 mL). After incubation (24 h, 37°C, 5% CO_2_ atmosphere), cultures were centrifuged (15 min, 3000 *×g*) and the supernatants were aspirated using sterile syringes and sterilised by filtration (0.22 μm nitrocellulose filter; Merck, Darmstadt, Germany) to obtain CFCS.

### Hydrophobicity assay

The hydrophobicity assay was adapted from Rodríguez et al. [58]. Suspensions (10^8^ CFU mL^-1^) of each strain were obtained as previously described, and the optical density (660 nm) was measured. The solvent (xylene, 0.4 mL) was added to each bacterial suspension (1 mL) and the mixtures were vortexed vigorously and incubated for 2 h at 37°C. Then, the lower aqueous phase was removed with subsequent measurement of the optical density. The percentage of hydrophobicity (H%) was calculated as follows: H% = ((A_0_ – A_2_) / A_0_) × 100, where A_0_ indicates the absorbance at time 0 h, and A_2_ is the absorbance after 2 h. Hydrophobicity can be presented as microbial adhesion to solvents (MATS), which are classified as hydrophilic (MATS ≤ 44.99%), amphiphilic (45.00% ≤ MATS ≤ 54.99%), or hydrophobic (MATS ≥ 55.00%) [59]. In the second classification, hydrophobicity can also be presented as microbial adhesion to hydrocarbons (MATH), which can be classified as high (MATH > 66%), medium (33% < MATH < 66%), or low (MATH < 33%) [17].

### Autoaggregation and co-aggregation assays

Autoaggregation and co-aggregation assays were adapted from Kos et al. [60]. For the autoaggregation assay, *L. plantarum* suspensions (10^8^ CFU mL^-1^) were obtained as previously described. Then, these suspensions were vortexed (10 s) and incubated for 5 h at room temperature (25°C). Absorbance (660 nm) was measured at time 0 h (A_0_) and after 5 h (A_5_). The percentage of autoaggregation (AA%) was calculated using the following formula: AA% = ((A_0_ – A_5_) / A_0_) × 100.

For the co-aggregation assay, *L. plantarum* and pathogen strains were grown (MRS or BHI broth, 24 h, 37°C, 5% CO_2_ atmosphere), centrifuged (15 min, 3000 *×g*), washed twice with sterile saline (0.9% NaCl), and resuspended to a final cell density (10^8^ CFU mL^-1^). Cell suspensions with mixed suspensions containing equal volumes (1 mL) of each *Lactobacillus* strain and pathogen strain were vortexed (10 s) and incubated for 4 h at 37°C. Absorbance (660 nm) was measured before and after the incubation. The percentage of co-aggregation (CA%) was calculated using the following formula: CA% = [(A_LAC_ + A_PAT_) / 2 – A_MIX_] / [(A_LAC_ + A_PAT_)] / 2], where A_LAC_ indicates the absorbance of the *Lactobacillus* strain, A_PAT_ indicates the absorbance of the pathogen strain, and A_MIX_ indicates the absorbance of the mixtures.

### Biofilm formation assay

The biofilm formation assay was adapted from Ouarabi et al. [61]. Initially, suspensions (10^8^ CFU mL^-1^) of *L. plantarum* strains were obtained as previously described. An aliquot (10 μL) of each *Lactobacillus* strain was inoculated separately in trypticase soy broth (TSB, Acumedia) (200 μL) supplemented with peptone (20 g mL^-1^) in a 96-well polystyrene plate and then incubated overnight. After incubation, plates were washed twice with sterile saline to remove non-adherent cells. Cells were fixed with 96% ethanol (200 μL) and incubated for 15 min at room temperature (25°C). Plates were emptied and then filled with violet crystals (200 μL, 0.1%) and incubated for 15 min at room temperature (25°C). The plates were then washed twice with sterile saline, and the wells were resuspended in 96% ethanol (200 μL); absorbance (650 nm) was immediately measured and used as an indication of biofilm formation. Sterile medium was included as a negative control to ensure that the influence on biofilm formation was not attributed to a non-specific binding effect to crystal violet. Based on the optical densities of the isolates (OD_I_) and the negative control (OD_C_), the formation of biofilms by *Lactobacillus* strains was classified according to their adherence: non-adherent, OD_I_ ≤ OD_C_; weakly adherent, OD_C_ < OD_I_ ≤ (2 × OD_C_), moderately adherent: (2 × OD_C_) < OD_I_ ≤ (4 × OD_C_); strongly adherent: (4 × OD_C_) < OD_I_.

### Haemolytic activity assay

The haemolytic activity assay was adapted from Abouloifa et al. [15]. Initially, suspensions (10^8^ CFU mL^-1^) of *L. plantarum* strains were obtained as previously described and were spot-inoculated (10 μL) on 5% human blood agar. After incubating the plates (48 h, 37°C, 5% CO_2_ atmosphere), haemolytic activity was detected by observing a clear zone of hydrolysis around the colonies (β-haemolysis), partial hydrolysis with green-hued zones around colonies (α-haemolysis), or no zone around colonies (γ-haemolysis). γ-Haemolysis was considered negative haemolysis.

### Antibiotic susceptibility assay

The susceptibility of *Lactobacillus* strains to antimicrobials was determined by the modified disk-diffusion method of the Clinical and Laboratory Standards Institute (CLSI). Overnight cultures of *Lactobacillus* strains were adjusted to 0.5 McFarland standards. An aliquot of this suspension was then swabbed onto MRS agar plates, followed by the arrangement of antibiotic disks. The antimicrobials (Laborclin, Pinhais, SP, Brazil) tested were ampicillin (10 μg), ceftriaxone (30 μg), ciprofloxacin (5 μg), clindamycin (2 μg), chloramphenicol (30 μg), erythromycin (15 μg), nitrofurantoin (300 μg), penicillin (10 μg) and vancomycin (30 μg). Plates were incubated overnight, and the diameters of the halos were measured and classified as sensitive (S), susceptible, increased exposure (SIE), and resistant (R), according to Charteris et al. [62]. *Staphylococcus aureus* ATCC 25923 was used as the positive control.

### Heat tolerance assay

The heat resistance of *Lactobacillus* strains was evaluated according to Paéz et al. [35], with modifications. Initially, suspensions (10^8^ CFU mL^-1^) of *L. plantarum* were obtained as previously described. An aliquot (100 μL) was resuspended in volume (500 μL) of 10% skim milk (Nestlé, Araçatuba, SP, Brazil). Then, each cell suspension was incubated in a water bath (60°C, 5 min), followed by cooling in an ice bath. Aliquots (10 μL) of each strain were plated on MRS agar, and after incubation (48 h, 37°C, 5% CO_2_ atmosphere) colonies were counted and enumerated considering CFU mL^-1^. As a control, aliquots (10 μL) of the same samples were plated under the same conditions before exposure to heat.

### pH tolerance assay

Analysis of bacterial growth under various pH conditions was adapted from Melo et al. [16]. MRS and BHI broth solutions of pH 3-8 were prepared by addition of 1 mol L^-1^ of hydrochloric acid or sodium hydroxide. Before the assay, suspensions (10^8^ CFU mL^-1^) of each strain (*Lactobacillus* and pathogens) were obtained as previously described. Trials were performed in 96-well microplates, where 180 μL of MRS or BHI broth at each pH was inoculated with 20 μL of active culture or saline as a control. The microplate was incubated overnight, and the optical density (600 nm) was determined at 8 h-intervals using a spectrophotometer (Tp-reader, Thermoplate, USA).

### Coculture inhibition assay

The antimicrobial activity of *Lactobacillus* strains against pathogens was tested using a co-culture assay adapted from Hütt et al. [63]. Initially, suspensions (10^8^ CFU mL^-1^) of *Lactobacillus* and pathogens were obtained as previously described. Activated cultures of pathogens and *Lactobacillus* were inoculated together (1%, v/v) in mixed growth medium (0.5 mL BHI broth + 0.5 mL MRS broth) and incubated (18 to 24 h, 37°C, 5% CO_2_ atmosphere). Serial dilutions were performed and aliquots (10 μL) were seeded on blood or chocolate agar followed by the reincubation (24 h, 37°C, 5% CO_2_ atmosphere) of plates. Cultures performed with the pathogen alone were used as negative controls. The growth of the pathogen with each *Lactobacillus* strain was compared with the growth of the control.

### Evaluation of pH modulation by *Lactobacillus* strains

The ability of *Lactobacillus* strains to modulate the pH of the growth medium with or without pathogens was evaluated according to Melgaço et al. [64], with modifications. This test separately evaluated the modulation of pH with cultures isolated from lactobacilli strains in MRS medium, the modulation of pH with mixed cultures of each *Lactobacillus* strain with *G. vaginalis* in MRS + BHI medium, and the modulation of pH with mixed cultures from each *Lactobacillus* strain with *N. gonorrhoeae*. Initially, microorganism suspensions (10^8^ CFU mL^-1^) were obtained as previously described and the pH of the MRS or MRS + BHI (v/v) broths was measured and adjusted to 6.5. Then, an aliquot of each *Lactobacillus* strain was added separately to the broth (10%, v/v) and the same volume of pathogen strains was added separately to the MRS + BHI broth. After incubation (24 h, 37°C, 5% CO_2_ atmosphere), cultures were centrifuged (15 min, 3000 *×g*), the bacterial pellet was separated from the supernatant, and the pH of the bacterial cultures was measured (HMMPB-210, Highmed, Tatuapé, SP, Brazil).

### Determination of CFCS antimicrobial activity: Deferred inhibition assay and microdiffusion assay on semi-solid agar

Antimicrobial activity was evaluated by the deferred inhibition assay according to Nardi et al. [65]. Initially, an aliquot (5 μL) of each lactobacillus strain suspension (10^8^ CFU mL^-1^) was pipetted into the centre of the plate with MRS agar. After incubation for 48 h at 37°C in a 5% CO_2_ atmosphere, colony cells were killed by exposure to chloroform (30 min, 1 mL). Residual chloroform was evaporated and the Petri dish was overlaid with BHI semi-solid agar (3.5 mL, 0.75%, w/v), previously inoculated with pathogens (1%, v/v, 10^8^ CFU mL^-1^). After overnight incubation, there was an inhibition halo. After incubation (18-24 h, 37°C, 5% CO_2_ atmosphere), the presence or absence of inhibition halos was observed, followed by measuring the inhibition halos (mm). Sterile MRS broth was used as the negative control.

The presence of diffusible inhibitory substances was also evaluated by a microdiffusion assay on semi-solid agar adapted from Rodrigues et al. [66]. Initially, suspensions of pathogens (10^8^ CFU mL^-1^) were added (1%, v/v) on semi-solid BHI agar (0.75%, w/v) and plated. After solidification, sterile polyvinyl chloride (PVC) cylinders (8 mm) were placed centrally on the plates and aliquots (100 μL) of CFCS from each *Lactobacillus* were added. After incubation (18-24 h, 37°C, 5% CO_2_ atmosphere), the presence or absence of inhibition halos was observed, followed by measurement of the inhibition halos (mm). Sterile MRS broth was used as the negative control.

### Detection of organic acids, thermotolerant antimicrobial substances, and bacteriocins in the CFCS

The lactobacilli strains were assayed for the production of organic acids, thermotolerant antimicrobial substances, and bacteriocins using the agar-well diffusion technique described by Touré et al. [67], with modifications. Initially, *G. vaginalis* suspension (10^8^ CFU mL^-1^) was swabbed onto 5% blood agar plates, and *N. gonorrhoeae* suspension (10^8^ CFU mL^-1^) was swabbed onto chocolate agar plates. Plates were then incubated for 30 min at room temperature (25°C). Concomitantly, the CFCS aliquots were distributed in fractions for treatment. For the organic acid assay, the CFCS was adjusted to pH 6.5 ± 0.1, using 1 mol · L^-1^ sodium hydroxide; for the thermotolerant substance assay the CFCS was incubated at high temperature (5 min, 100°C), and for bacteriocin assay the CSCF was treated with trypsin (1%, v/v; Gibco, Mississauga, Canada) or proteinase K (1%, v/v, Invitrogen, Darmstadt, Germany). Aliquots (100 μL) of treated and untreated CFCS were added to the wells (8 mm diameter) previously made on chocolate and 5% blood agar plates. Plates were incubated overnight and the diameters of the inhibition zones (including the 8 mm well diameter) were measured.

### Amplex red hydrogen peroxide assay

Hydrogen peroxide levels present in CFCS were measured using the Amplex Red Hydrogen Peroxide/Peroxidase kit according to the manufacturer’s recommendations (Thermo Fisher Scientific, Waltham, MA, USA). After preparing the kit stock solutions, aliquots (50 μL) of the standard curve samples, controls, and experimental samples were added to individual wells on a microplate. The Amplex Red reagent/HRP working solution (50 μL) was added to the wells previously plotted. After incubation (30 min, room temperature - 25°C, protected from light), the absorbance was measured in a microplate reader (550 nm) to construct the standard curve and measure the H_2_O_2_ concentration (μM) of the CFCS.

### Analysis of the CFCS metabolome by GC-MS

The CFCS metabolome was analysed by gas chromatography-mass spectrometry (GC-MS) according to the method described by Rodrigues et al. [66]. Initially, the CFCS of each *Lactobacillus* strain was previously conditioned (48 h, -18°C), then the frozen samples were inserted in a freeze dryer (Alpha 1-2 LDplus, CHRIST, Osterode, Germany) and subjected to the sublimation drying process (48 h) in two distinct phases. The primary drying phase consisted of removing free water (-20°C; 1.0 mbar) and the secondary drying phase consisted of partial removal of bound water (-30°C; 0.34 mbar). At the end of the process, the lyophilized CFCS was derivatized by silylation, and lyophilized samples (3 mg) were diluted in a mixture of 100 μL *N, O-bis* (trimethylsilyl)trifluoroacetamide (BSTFA) containing 1% trimethylchlorosilane (Sigma-Aldrich, Merck, Darmstadt, Germany) with pyridine (60 μL). During this reaction, mixtures were incubated for 30 min at 70°C in a water bath for better dilution. Samples were then injected (1 μL) separately into the chromatograph (QP2010SE-GC2010 Plus, Shimadzu, Kyoto, Japan) for metabolome screening. The hardware and software configurations of the equipment are described below: Chromatograph with Rtx-5MS (0.25 μm film, 30 m, and 0.25 mm internal diameter); helium gas as carrier gas; temperature of 290°C used in the injector, in the detector, and in the GC-MS system interface; initial temperature of 80°C (5 min); final temperature of 285°C (20 min); gradual increase from initial temperature to final temperature of 4°C min^-1^; the sweep mass operated from 30 to 600 Da; and the mass detector operated with electron impact ionisation (70 eV). GC-MS identified the substances present in the CFCS when comparing the mass spectra existing in the equipment database (WILEY8, NIST 08, and FFNSC1.3) with the mass spectra of the CFCS samples. This chromatography analysis did not require the use of positive or negative controls.

### Statistical analysis

GraphPad Prism 6.0 software (GraphPad Software, San Diego, CA, USA) was used for statistical analysis. Quantitative data are presented by means and standard deviations. Normality was tested by D'Agostino & Pearson, Shapiro-Wilk and KS tests. The statistical differences between mean values were determined by the *t* test, Mann-Whitney test or Kruskal-Wallis test with Dunn's post-test. Data were considered statistically significant when: * = *P* < 0.05, ** *P* < 0.01, *** = *P* < 0.001, **** = *P* < 0.0001. Except for CFCS metabolome, all assays were performed in triplicate.

## Data Availability

The datasets used and/or analysed during the current study are available from the corresponding author upon reasonable request.

## Abbreviations

AA%: Percentage of autoaggregation
ATCC: American Type Culture Collection
BHI: brain and heart infusion
BSTFA: *N,O*-Bis(trimethylsilyl)trifluoroacetamide
BV: bacterial vaginosis
CA%: percentage of co-aggregation
CFCS: cell-free culture supernatants
CLSI: Clinical and Laboratory Standards Institute
FAO: Food and Agriculture Organization
GC-MS: Gas chromatography-mass spectrometry
GRAS: Generally recognised as safe
H%: Percentage of hydrophobicity
Lp03: *Lactiplantibacillus plantarum* 03
Lp289: *Lactiplantibacillus plantarum* 289
Lp291: *Lactiplantibacillus plantarum* 291
MATH: Microbial adhesion to hydrocarbons
MATS: Microbial adhesion to solvents
MRS: Man, Rogosa, and Sharpe
OD: Optical density
PVC: Polyvinyl chloride
TSB: Trypticase soy broth
